# Reproducibility of the Time of Intraocular Pressure Peaks During Water-drinking Test in Patients Treated for Open-angle Glaucoma

**DOI:** 10.18502/jovr.v19i4.14982

**Published:** 2024-12-31

**Authors:** Carolina TN Susanna, C. Gustavo De Moraes, Paula Alhadeff, Bianca TN Susanna, Fernanda TN Susanna, Renato Antunes Schiave Germano, Remo Susanna Jr

**Affiliations:** ^1^Department of Ophthalmology, University of Sao Paulo School of Medicine, Sao Paulo, Brazil; ^2^Department of Ophthalmology, Columbia University Irving Medical Center, New York, NY, USA; ^3^Centro de Otalmologia Especializado (COE), S ao Paulo, S ao Paulo, Brazil; ^4^Banco de Olhos de Sorocaba (BOS), Sorocaba, S ao Paulo, Brazil; ^6^Carolina Nicolela Susanna: https://orcid.org/0000-0003-1341-6112; ^7^Remo Susanna Jr: https://orcid.org/0000-0001-9147-9528

**Keywords:** Glaucoma Severity, IOP Peak Time, Primary Open-angle Glaucoma, Reproducibility, Water Drinking Test

## Abstract

**Purpose:**

To evaluate the reproducibility and intra-eye similarity of the intraocular pressure (IOP) peaks induced by the water drinking test (WDT) in treated glaucoma patients.

**Methods:**

This prospective cohort study evaluated 99 patients (198 eyes) who were treated for primary open-angle glaucoma. All patients underwent WDT in two consecutive visits with no change in their current therapy. The interval between the tests was 4 four to six months. The tests were administered at a similar time (4:00 PM ± 1 hour). The reproducibility of the time of the IOP peaks and the correlation between the peak time of both eyes during the two consecutive WDT sessions were assessed.

**Results:**

Of all IOP peaks, 59.6% and 71.7% occurred at the same time during the two WDT sessions in the right and left eyes, respectively. In the first and second WDT sessions, the agreements in IOP peak time between the right and left eyes were 60% and 63%, respectively.

**Conclusion:**

The IOP peak time between the two consecutive WDT sessions was moderately reproducible, and there was a moderate agreement in the peak time between the two eyes. In light of these findings, clinicians should avoid performing simplified versions of WDT to evaluate IOP peaks.

##  INTRODUCTION

Elevated intraocular pressure (IOP) is widely acknowledged as the leading risk factor for the initiation and advancement of glaucoma.^[1,2,3]^ Current treatment approaches aim to reduce IOP to a target level where additional damage is less likely to occur. Since an elevation of 1 mmHg in IOP correlates with a 10% rise in the relative risk of glaucoma development^[1]^ and visual field (VF) deterioration^[3,4]^, reducing IOP is the mainstay of glaucoma management. Even with seemingly well-controlled IOP levels, some patients continue to experience glaucoma progression. While some researchers have suggested that this may be due to IOP fluctuations,^[5,6,7]^, recent studies have shown that IOP peak can better predict glaucoma progression.^[8,9,10,11,12,13,14,15]^ However, IOP peak is not routinely assessed in clinical practice. Continuous 24-hour IOP monitoring could theoretically offer better insights into an individual's IOP and peak pressure, but it is impractical in clinical settings due to its time- and resource-intensive nature. The modified diurnal tension curve, also known as phasing, is an alternative that involves taking four to five IOP measurements during working hours (from 8 am to 6 pm). However, IOP peaks most often occur outside of office hours.^[16]^

The water drinking test (WDT) can serve as an indirect marker for outflow facility and can predict 24-hour IOP peaks. As shown by several studies, peaks induced by WDT strongly correlate and agree with IOP peaks occurring throughout the day.^[17,18,19]^ The WDT is also associated with the risk of glaucomatous VF progression and disease severity,^[11,20,21,22]^ and is considered an indicator of treatment efficacy.^[23,24,25,26,27]^ Moreover, a recent study has shown that the time of the IOP peak correlates with the level of glaucoma damage. In fact, eyes with reduced outflow facility and more advanced glaucoma defects may experience sustained IOP increases during WDT, leading to later IOP peaks (30–45 minutes after baseline) than eyes with better outflow facility.^[28]^

Our study aims to (i) evaluate the reproducibility of the time of the IOP peak during WDT on different days and (ii) assess whether both eyes experience IOP peaks during WDT at the same time (here called IOP peak symmetry).

##  METHODS

This prospective cross-sectional study included 198 eyes from 99 participants. The study adhered to the principles of the Declaration of Helsinki and was approved by the ethics committee at Hospital das Clínicas da Faculdade de Medicina da USP in Brazil (approval no. 75914323.8.0000.0068). All participants provided their written informed consent. Eligible patients were enrolled consecutively based on the predefined criteria for inclusion and exclusion.

Patients underwent a comprehensive review of their medical history, IOP measurement using Goldmann applanation tonometry, evaluation of best-corrected visual acuity (BCVA), and slit-lamp biomicroscopy. The inclusion criteria required the optic disc to have a glaucomatous appearance, as confirmed by a senior glaucoma specialist through disc photograph evaluation, along with glaucomatous VF loss on 24-2 standard automated perimetry. VF loss was defined according to the modified Hodapp-Parrish-Anderson criteria and was confirmed in at least two consecutive examinations.^[28]^

The eyes included in the study had a minimum BCVA of 20/40, spherical refraction of up to ± 5.00 diopters (D), and cylinder correction of up to 3.00 D. Participants were excluded if they presented with closed or narrow angles (determined by gonioscopy), non-glaucomatous optic neuropathy, retinal disease, secondary glaucoma, or any other conditions that could potentially impact VF testing. None of the patients had undergone laser trabeculoplasty, trabeculectomy, or cataract surgery within six months prior to enrollment.^[28]^

Treatment options during the study could be freely chosen by the principal clinician, yet they had to stay the same during the first and second sessions of WDT to prevent bias.^[28]^

During WDT, the initial IOP measurement served as the baseline before participants drank 800 mL of water within 5 minutes. Subsequent IOP measurements were taken three times at 15-minute intervals. Participants were instructed to refrain from drinking liquids for at least 2 hours prior to the test. IOP was assessed using a Goldmann applanation tonometer (Haag-Streit, GmbH, Switzerland). The highest recorded value among the three measurements was identified as the peak IOP during WDT. Peak time was defined as the point when maximum pressure was recorded. All WDTs were conducted between 4:00 PM and 5:00 PM to minimize the influence of circadian variations on IOP. Data from both eyes were included based on predefined inclusion and exclusion criteria.^[28]^

All selected patients underwent WDT on two different days (WDT1 and WDT2) to allow evaluating the reproducibility of IOP peak time during WDT in those two days and assessing whether both eyes experienced IOP peaks simultaneously during WDT (IOP peak symmetry).

### Statistical Analysis

Descriptive statistics involved measuring center (mean) and dispersion (SD). The main outcome was the proportion of eyes (%) in which the IOP peak time coincided between the two WDT sessions. Data from the right and left eyes were presented separately. For the secondary outcomes, we assessed how often (%) the time of the IOP peak in the right and left eyes coincided. We also tested the association (Pearson correlation test) and agreement (Cohen kappa) between the times of IOP peaks between the two sessions. Lastly, the differences between IOP peak values were presented using Bland-Altman plots. Statistical significance was set at *P* < 0.05. Computerized statistical analyses were conducted using Stata version 14.2 (Stata Corp, Texas, USA).

**Table 1:** Baseline characteristics.


**Demographic characteristics**	
Age	66.53 ± 12.65 ${\;}^{*}$
Sex	
• Male	44 (44.44%)
• Female	55 (55.56%)
Race	
• White	93 (93.94%)
• Asian	6 (6.06%)
MD	
• OD	–4.34 ± 5.75 ${\;}^{*}$
• OS	–5.37 ± 6.38 ${\;}^{*}$
IOP baseline #1	
• OD	12.44 ± 2.57 ${\;}^{*}$
• OS	12.24 ± 2.54 ${\;}^{*}$
IOP peak #1	
• OD	14.94 ± 2.70 ${\;}^{*}$
• OS	15.07 ± 2.77 ${\;}^{*}$
IOP baseline #2	
• OD	12.30 ± 2.76 ${\;}^{*}$
• OS	12.38 ± 2.80 ${\;}^{*}$
IOP peak #2	
• OD	15.20 ± 2.97 ${\;}^{*}$
• OS	15.39 ± 3.00 ${\;}^{*}$
Number of medications	
• OD	1.96 ± 1.33 ${\;}^{*}$
• OS	2.07 ± 1.31 ${\;}^{*}$
Types of medications, OD	
• Prostaglandin	64 (64.65%)
• Beta-blocker	65 (65.66%)
• Alpha-adrenergic	14 (14.14%)
• Carbonic anhydrase inhibitor	49 (49.49%)
Types of medications, OS	
• Prostaglandin	67 (67.68%)
• Beta-blocker	70 (70.71%)
• Alpha-adrenergic	13 (13.13%)
• Carbonic anhydrase inhibitor	52 (52.53%)
	
	
${\;}^{*}$ Results are expressed in mean ± SD, calculated using summary statistics IOP, intraocular pressure; MD, mean deviation; OD, right eye; OS, left eye	

**Table 2:** Frequency (%) of IOP peaks for each time point for the right eyes.


**Time**	**WDT #1**	**WDT #2**
15 minutes	48 (48.5%)	47 (47.5%)
30 minutes	32 (32.3%)	31 (31.3%)
45 minutes	18 (18.2%)	20 (20.2%)
	
	
IOP, intraocular pressure; WDT, water-drinking test		

**Table 3:** Frequency (%) of IOP peaks for each time point for the left eyes.


**Time**	**WDT #1**	**WDT #2**
15 minutes	49 (49.5%)	51 (51.5%)
30 minutes	39 (39.4%)	40 (40.4%)
45 minutes	10 (10.1%)	8 (8.1%)
	
	
IOP, intraocular pressure; WDT, water-drinking test		

**Figure 1:**
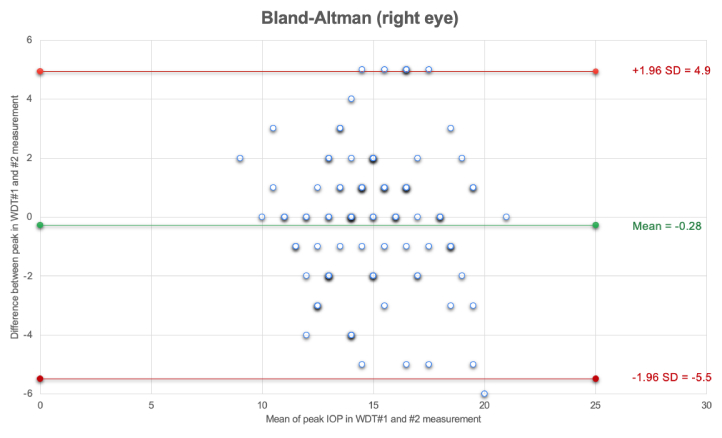
The Bland-Altman plots representing the agreement between IOP peaks in WDT1 (baseline) and WDT2 (post four to six months) for the right eyes.

**Figure 2:**
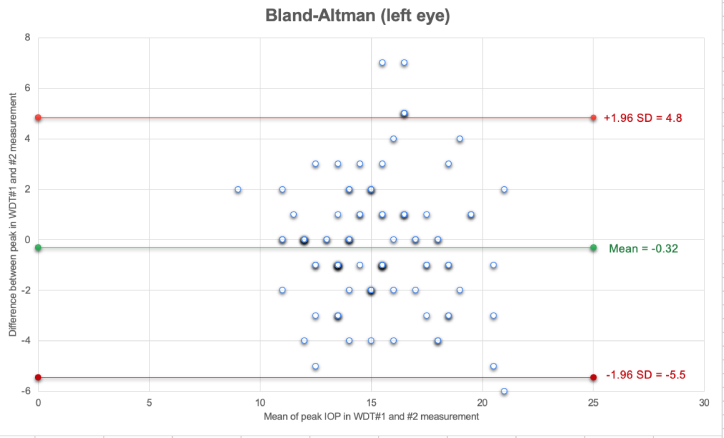
The Bland-Altman plots representing the agreement between IOP peaks in WDT1 (baseline) and WDT2 (post four to six months) measurement for the left eyes.

##  RESULTS

We analyzed 198 eyes of 99 patients receiving treatment for primary open-angle glaucoma (POAG). The mean (SD) age of the participants was 66.53 ± 12.65, and most of them were women (55.56%) and of European descent (93.94%). Table 1 summarizes the clinical characteristics of the patients. The frequency of IOP peaks for each time point is described in Tables 2 and 3 for the right and left eyes, respectively.

There was an overall modest agreement regarding the mean time of IOP peaks between the two WDT sessions (59.6% for the right eye and 71.7% for the left eyes; Cohen kappa = 0.36 and 0.51, respectively). Moderate correlation was also noticed between the time of IOP peaks (Pearson *r* = 0.43 and 0.46 for the right and left eyes, respectively).

The inter-eye similarity of IOP peaks was moderate between the two WDT sessions (60.6%% for the right eye and 63.6% for the left eye; Cohen kappa = 0.35 and 0.40, respectively). The intra-eye correlation was also moderate in the two WDT sessions (Pearson *r* = 0.41 and 0.50, respectively).

The Bland-Altman plots indicated IOP peak time (WDT1 and WDT2) had a mean difference of –0.28 ± 2.66 mmHg for the right eyes and –0.32 ± 2.66 mmHg for the left eyes.

##  DISCUSSION 

Multiple studies have demonstrated the clinical significance and utility of WDT in managing POAG. This test has been employed to compare the effects of various clinical and surgical treatments for glaucoma. Even with similar baseline mean IOPs, patients with medically controlled glaucoma exhibit a greater IOP increase during WDT compared to those who have undergone filtration procedures such as deep sclerectomy or trabeculectomy.^[27,30,31]^ This difference may be attributed to the fact that filtration procedures facilitate aqueous humor outflow more effectively than medications. This rationale can also be applied to evaluate the effectiveness of different hypotensive glaucoma drops. For instance, in a study comparing latanoprost and a fixed combination of timolol and dorzolamide, patients receiving latanoprost showed significantly lower IOP elevations following WDT, despite similar IOP reductions.^[32]^ In fact, the ability to reduce IOP peaks might be an additional benefit of the prostaglandins.

Both severity and progression of glaucoma are linked to higher IOP peaks. In a cohort of patients with POAG and asymmetric VF defects, the eyes with worse mean deviation (MD) values had higher IOP peaks after water ingestion compared to their contralateral eyes with better VF, despite similar mean IOPs at baseline. This study illustrated that eyes with more glaucoma-related damage had a reduced capacity to control IOP.^[22]^ Another study found that the mean IOP peak and the percentage of IOP variation during the test were significantly higher in patients with VF loss than those with no progression, despite similar baseline IOPs.^[33]^

In addition to the value of IOP peak, the timing of the IOP peak is also associated with the extent of glaucoma damage. A later peak time during the test is linked to more severe glaucomatous damage, because eyes with poorer outflow facility may experience continued IOP elevation during WDT, resulting in later IOP peaks than eyes with better outflow facility.^[28]^ Likewise, De Moraes et al concluded that the number of prolonged peaks assessed with a contact lens sensor is the best predictor of faster progression of glaucoma.^[34]^

A test must produce consistent and reproducible results to be deemed clinically significant. Given the irreversibility of glaucomatous damage, it is essential to estimate the risk of progression early before it occurs since greater damage increases the risk of blindness and other disabilities associated with glaucoma progression. Hatanaka et al demonstrated that performing WDT at the same time on two consecutive days resulted in highly reproducible IOP peak values.^[35]^ Similarly, consistent reproducibility of IOP peaks was noted when the test was conducted four months apart at the same time of day.^[36]^

The use of WDT is still limited, despite extensive evidence linking IOP peak to glaucoma progression and the cost-effectiveness and feasibility of WDT compared to other tests for IOP peak estimation. One reason is that this test is considered time-consuming in busy clinics. To deal with this issue, some ophthalmologists ask the patient to do the WDT at home using the iCare Home device. This could obviate the need for the clinic to keep the patient for 45 minutes and have a technician or physician repeatedly measure the IOP. Alternatively, some clinicians ask patients to drink 800 ml of water within 5 minutes 30 to 40 minutes before the eye examination, as most IOP peaks in moderate and severe glaucoma occur during this time frame.^[28]^ However, to validate this approach, it is necessary to establish whether IOP peaks occur at the same time during the test on different days.

In a study by Xu et al,^[37]^ 24-hour IOP curves in untreated patients with POAG and ocular hypertension, only 37.23% and 35.29% of IOP peak points occurred within a 2-hour difference in patients with POAG and ocular hypertension, respectively. Realini et al^[38,39]^ similarly showed that neither healthy individuals nor patients with treated POAG manifested a sustained diurnal IOP pattern during office hours (from 8 am to 8 pm). In agreement with these studies, our study found a modest reproducibility in the time of IOP peaks in consecutive WDT sessions, with the peak IOP occurring simultaneously in the right and left eyes in 59% and 71% of cases, respectively. This modest reproducibility may be related to the time when WDT is performed. A minimal rise and an earlier IOP peak are expected if the test is performed during or closer to the peak diurnal tension. As stated by Miller, each eye probably has an area of upper limit of tension above which it will not override.^[40]^

The agreement in IOP peak times between the two eyes was only 60% and 63% in the first and second WDT, respectively. This result is not unexpected as glaucoma is an asymmetrical disease. Susanna et al demonstrated that the time IOP peak occurs during WDT may be correlated with the severity of both outflow impairment and glaucoma.^[28]^ Therefore, while peak IOP during WDT is a valuable clinical parameter, caution should be taken when interpreting IOP peak values at a particular time point.

One limitation of our study is that the majority of patients were White, with few oriental and no Afro-descendent patients. Therefore, the results have to be interpreted with caution when applied to the IOP characteristics of other populations and other methods of assessment.

In summary, our study demonstrated a modest reproducibility in the time of IOP peaks between two consecutive WDT sessions and a moderate agreement in the time of peaks between the two eyes. Given the variability in IOP peak timing and the potential for misinterpretation, clinicians should exercise caution when using simplified versions of WDT.

##  Financial Support and Sponsorship

None.

##  Conflicts of Interest

None.
